# Establishment of an In Vitro Disease Model of Charcot-Marie-Tooth Disease using Human Induced Pluripotent Stem Cells

**DOI:** 10.14789/ejmj.JMJ24-0046-R

**Published:** 2025-05-09

**Authors:** XING LIU, KEI-ICHI ISHIKAWA, NOBUTAKA HATTORI, WADO AKAMATSU

**Affiliations:** 1 Center for Genomic and Regenerative Medicine, Juntendo University, Tokyo, Japan; 1 Center for Genomic and Regenerative Medicine, Juntendo University, Tokyo, Japan; 2 Department of Neurology, Juntendo University, Faculty of Medicine, Tokyo, Japan; 2 Department of Neurology, Juntendo University, Faculty of Medicine, Tokyo, Japan; 3 Department of Research and Development for Organoids, Juntendo University School of Medicine, Tokyo, Japan; 3 Department of Research and Development for Organoids, Juntendo University School of Medicine, Tokyo, Japan; 4 Neurodegenerative Disorders Collaborative Laboratory, RIKEN Center for Brain Science, Saitama, Japan; 4 Neurodegenerative Disorders Collaborative Laboratory, RIKEN Center for Brain Science, Saitama, Japan

**Keywords:** iPSC, Charcot-Marie-Tooth disease, PMP22, Schwann cell, peripheral neuropathy

## Abstract

Charcot-Marie-Tooth disease type 1A (CMT1A) is a hereditary neuropathy caused by the duplication of the PMP22 gene, leading to Schwann cell dysfunction and peripheral demyelination. We developed a Schwann cell lineage model derived from induced pluripotent stem cells (iPSCs) obtained from a CMT1A patient. This model exhibited disease-specific phenotypes, providing a valuable platform for investigating the pathophysiology of CMT1A and exploring therapeutic strategies.

Charcot-Marie-Tooth disease (CMT) describes a group of inherited neurological disorders that affect peripheral sensory and motor neurons, leading to progressive muscle weakness, atrophy, and sensory loss, especially in distal limbs. Duplication of *PMP22*, one of the most frequently observed CMT pathogenic mutations, is believed to contribute to Schwann cell dysfunction and demyelination. The molecular mechanism by which *PMP22* duplication causes demyelination has yet to be elucidated and no effective treatments aside from supportive care has been established. Developing an in vitro disease model using induced pluripotent stem cells (iPSCs), that reflect patient genotypes may provide valuable insights into the pathology caused by *PMP22* duplication. Thus, this study aims to establish an iPSC-derived disease model of CMT1A.

iPSCs were established from T cells of a CMT1A patient by transducing Yamanaka factors (Klf4, Oct3/4, Sox2, c-Myc) using Sendai virus vectors. Cell quality and identity were verified in our previous report^1)^. The Juntendo University School of Medicine Ethics Committee approved all experiments for human iPSCs (Approval number: 2020074).

To understand the pathophysiology of CMT, CMT1A-iPSCs were differentiated into Schwann cells according to a previously reported protocol^2)^. Variability of Schwann cell precursors (SCP) was confirmed across passages and after freeze-thaw cycles, indicating that the SCPs generated by this protocol are expandable and can be cryopreserved. Both SCPs and SCs displayed corresponding morphologies. qPCR and immunochemical staining confirmed the expression of Schwann cell precursor markers (SOX10, NGFR, GAP43) and mature Schwann cell markers (MPZ, GAP43, S100β). Notably, an elevation of *PMP22* expression in patient-derived SCPs and SCs was observed compared to healthy control (Figure 1).

**Figure 1:**
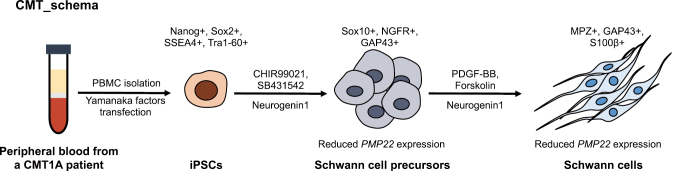
Schematic representation of the establishment of in vitro CMT1A disease model CMT1A: Charcot-Marie-Tooth disease type 1A; PBMC: peripheral blood mononuclear cell; iPSC: induced pluripotent stem cell; PDGF-BB: platelet derived growth factor BB

In summary, this study established and validated a stable and expandable iPS cell line from a CMT1A patient. Then CMT1A-iPSCs were differentiated into the SCP-SC lineage and successfully expressed disease-specific phenotypes. This model is a promising tool for drug screening and further elucidation of CMT1A pathophysiology. Although several other iPSC-derived CMT1A models have been reported, current studies have focused more on neural crest cells or SCPs rather than mature SCs^3-6)^. Besides, in vitro studies of dysmyelination and demyelination, the essential pathological features of demyelinating CMT, are rarely reported due to the limited maturation of differentiated SCs and the low efficiency of in vitro myelination. Thus, establishing a stable in vitro myelination model could provide deeper insight into the disrupted interaction between SCs and peripheral neurons in CMT.

## Funding

This work is funded by Grant-in-Aid for Special Research in Subsidies for ordinary expenses of private schools from the Promotion and Mutual Aid Corporation for Private Schools of Japan.

## Author contributions

XL, KI, and WA conceived and designed the experiments. XL and KI performed the experiments and analyzed the data. XL, KI, NH, and WA wrote and revised the manuscript. All authors have reviewed and approved the manuscript.

## Conflicts of interest statement

The authors declare that there are no conflicts of interest.
